# The Neuroactive Steroid Pregnanolone Glutamate: Anticonvulsant Effect, Metabolites and Its Effect on Neurosteroid Levels in Developing Rat Brains

**DOI:** 10.3390/ph15010049

**Published:** 2021-12-30

**Authors:** Eva Kudova, Pavel Mares, Martin Hill, Katerina Vondrakova, Grygoriy Tsenov, Hana Chodounska, Hana Kubova, Karel Vales

**Affiliations:** 1 Institute of Organic Chemistry and Biochemistry of the Czech Academy of Sciences, Flemingovo namesti 2, 16000 Prague, Czech Republic; hchod@uochb.cas.cz; 2 Institute of Physiology, Academy of Sciences of the Czech Republic, Videnska 1083, 14220 Prague, Czech Republic; pavel.mares@fgu.cas.cz (P.M.); katavondrakova@seznam.cz (K.V.); hana.kubova@fgu.cas.cz (H.K.); karel.vales@nudz.cz (K.V.); 3 Institute of Endocrinology, Narodni 8, 11694 Prague, Czech Republic; mhill@endo.cz; 4 Institute of Mental Health, Topolova 748, 25067 Klecany, Czech Republic; 5 Department of Neurosciences, Biomedicine and Movement Sciences, University of Verona, Strada le Grazie 8, 37134 Verona, Italy

**Keywords:** neurosteroids, anticonvulsant, zuranolone, NMDA, GABA, metabolomics

## Abstract

Pregnanolone glutamate (PA-G) is a neuroactive steroid that has been previously demonstrated to be a potent neuroprotective compound in several biological models in vivo. Our in vitro experiments identified PA-G as an inhibitor of *N*-methyl-*D*-aspartate receptors and a potentiator of γ-aminobutyric acid receptors (GABA_A_Rs). In this study, we addressed the hypothesis that combined GABA_A_R potentiation and NMDAR antagonism could afford a potent anticonvulsant effect. Our results demonstrated the strong age-related anticonvulsive effect of PA-G in a model of pentylenetetrazol-induced seizures. PA-G significantly decreased seizure severity in 12-day-old animals, but only after the highest dose in 25-day-old animals. Interestingly, the anticonvulsant effect of PA-G differed both qualitatively and quantitatively from that of zuranolone, an investigational neurosteroid acting as a potent positive allosteric modulator of GABA_A_Rs. Next, we identified 17-hydroxy-pregnanolone (17-OH-PA) as a major metabolite of PA-G in 12-day-old animals. Finally, the administration of PA-G demonstrated direct modulation of unexpected neurosteroid levels, namely pregnenolone and dehydroepiandrosterone sulfate. These results suggest that compound PA-G might be a pro-drug of 17-OH-PA, a neurosteroid with a promising neuroprotective effect with an unknown mechanism of action that may represent an attractive target for studying perinatal neural diseases.

## 1. Introduction

Neurosteroids (NS) are synthesized de novo in the brain and modulate the receptors for neurotransmitters, especially glutamate and γ-aminobutyric acid (GABA_A_R). Both NS and their synthetic analogs, neuroactive steroids (NAS), readily cross the blood–brain barrier. The unconjugated structures are transported passively due to their lipophilic structure, while the conjugated steroids (e.g., sulfates) are dependent on selective uptake or efflux proteins [1,2,3]. Consequently, NS and NAS modulate many functions of the central and peripheral nervous system, including development and other complex behavior [4].

Interestingly, the levels of NS are altered during development [5] and aging [6], and under pathological conditions [7]. For example, the synaptic receptors for γ-aminobutyric acid (GABA_A_Rs) expressed in murine cortical pyramidal neurons and interneurons during early neonatal development (P7–15) are influenced by an endogenous NS tone. The findings of neurosteroids’ influence on brain-region-dependent neurotransmission [5,8,9] suggest the possibility of augmenting low levels of endogenous NS with appropriate exogenous NAS. Recent results from preclinical and clinical studies suggested that NAS may be a novel class of drugs for the treatment of central nervous system (CNS) disorders, including epilepsy [10,11].

**Figure 1:**
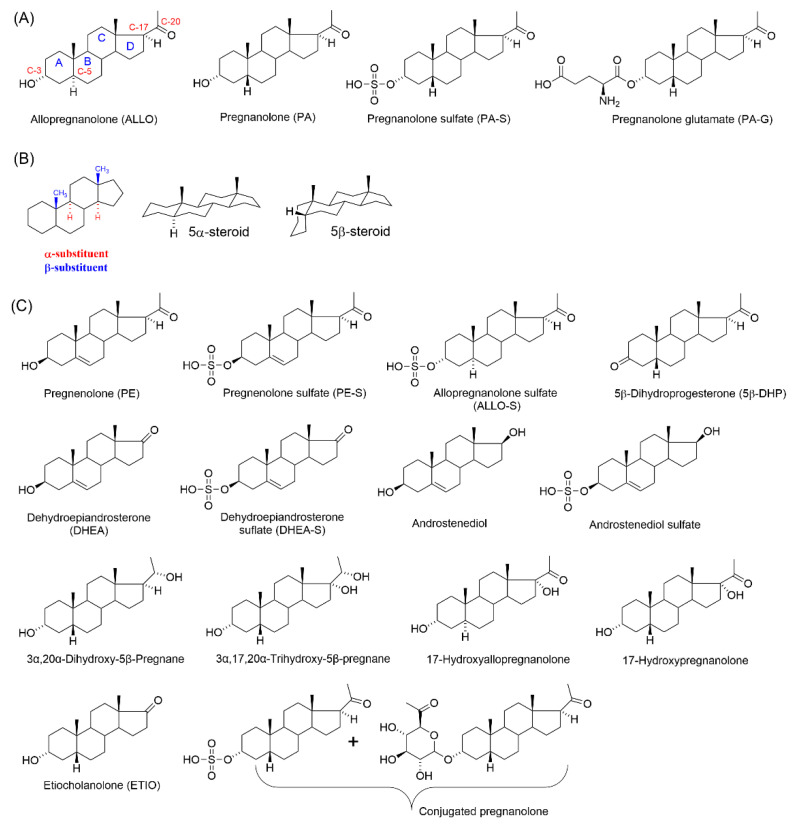
Structures of endogenous neurosteroids and neuroactive steroids relevant to this study. Steroidal ring numbering is in red; steroidal rings are in blue. (**A**) Structures of allopregnanolone, pregnanolone, pregnanolone sulfate, and pregnanolone glutamate. (**B**) Basics steroidal stereochemistry: the schematic orientation of the substituents and a perspective representation of a planar 5α steroid and a bent molecule of a 5β steroid. Note that when the rings of a steroid are denoted as projections onto the plane of the page, the α substituent (dashed bond) lies below it and the β substituent (bold bond) lies above the plane of the page. (**C**) Endogenous neurosteroids that were measured in the hippocampal tissue of rats after i.p. administration of PA-G.

The research studies usually only emphasize the GABAergic function, but there is a complex interaction between the neurotransmitter system and NS. For example, allopregnanolone (ALLO, Figure 1A), a positive modulator of GABA_A_Rs in nanomolar concentrations, is known to affect glutamate release via presynaptic GABA_A_Rs [12]. ALLO, its structural isomer pregnanolone (PA, Figure 1A), and their polar conjugates (i.e., sulfates or hemiesters) are effective modulators of GABA_A_Rs and *N*-methyl-*D*-aspartate receptors (NMDARs), influencing the permeability of ion channels [13,14,15]. The nature of positive or negative modulation depends on the combination of stereochemistry at position C-3 and/or the C-5 substituent (Figure 1B). For example, pregnane skeletons with 3α5α stereochemistry have a potent effect on GABA_A_Rs (i.e., ALLO), while 3α5β stereochemistry is typical of negative modulators of NMDARs. For example, the endogenous neurosteroid pregnanolone sulfate (PA-S, Figure 1A) shows a use-dependent mechanism of action on NMDARs such as memantine. In other words, the inhibitory effects are more expressed on glutamate-activated receptors [16]. Therefore, NAS augmentation may represent a strategy to maintain the excitation–inhibition balance of the brain during neurodevelopmental processes.

The concept of combined GABA_A_R potentiation and NMDAR antagonism has been previously described for the steroidal 3β-hydroxy neurosteroid MQ-221 [17] bearing an atypical stereochemistry for GABA_A_R potentiation and NMDAR inhibition. The authors proposed that MQ-221 may represent a new class of compounds for the treatment of neuropsychiatric disorders. This finding supports our hypothesis that targeting NMDARs and GABA_A_Rs with NAS may represent a strategy for treating epilepsy symptoms resulting from misbalanced inhibitory and excitatory neurotransmission.

NS that demonstrate significant protection against seizures in several animal models are known only as potent positive modulators of GABA_A_Rs, e.g., ALLO. The general concept of inhibitory action on NMDARs as a potential treatment for seizures has been widely reviewed in the literature [18,19]. In contrast, according to our knowledge, steroidal inhibitors of NMDARs as a potential anticonvulsant treatment have not been described. Therefore, we investigated the anticonvulsant profile of the previously well-described steroidal inhibitor of NMDARs, pregnanolone glutamate (PA-G, Figure 1A), at two developmental stages (postnatal days P12 and P25). As a comparison, endogenous PA-S was selected. Next, we performed a metabolomic study on samples of the hippocampus that were obtained after PA-G administration (i.p., 1 mg/kg) in male Wistar rats (P12 and P25). Finally, we measured the concentrations of basic endogenous NS after the administration of PA-G (Figure 1A,C).

## 2. Results

### 2.1. Anticonvulsant Effects of PA-S and PA-G in the Model of Pentylenetetrazol-Induced Seizures

**Figure 2:**
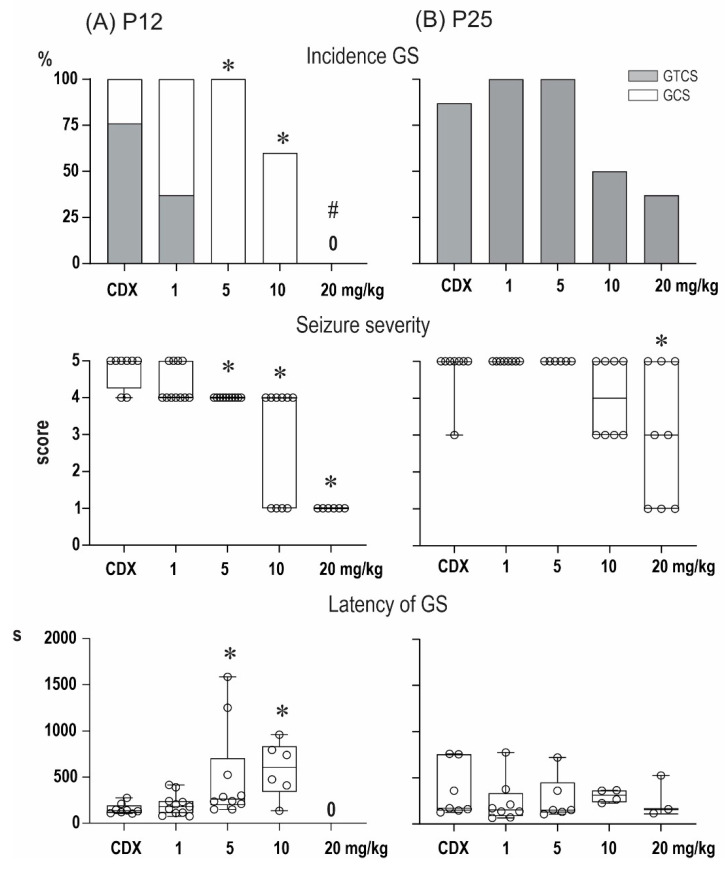
Effect of pregnanolone sulfate (PA-S) on seizures induced by pentylenetetrazol in 12- (**A**) and 25-day-old rats (**B**). From top to bottom: the incidence of generalized seizures (GS) is shown in columns (the black parts indicate complete generalized tonic-clonic seizures; the white parts show generalized clonic seizures without the tonic phase). The severity of seizures is expressed as a score [20], and the latencies of generalized seizures are shown as box plots (min to max) with individual values (circles). # denotes a significant difference in the incidence of GS compared with the controls, * denotes a significant difference in the incidence of TF in seizure severity and GS latency compared with the controls, and 0 means that none of the rats in the group exhibited GS.

**Figure 3:**
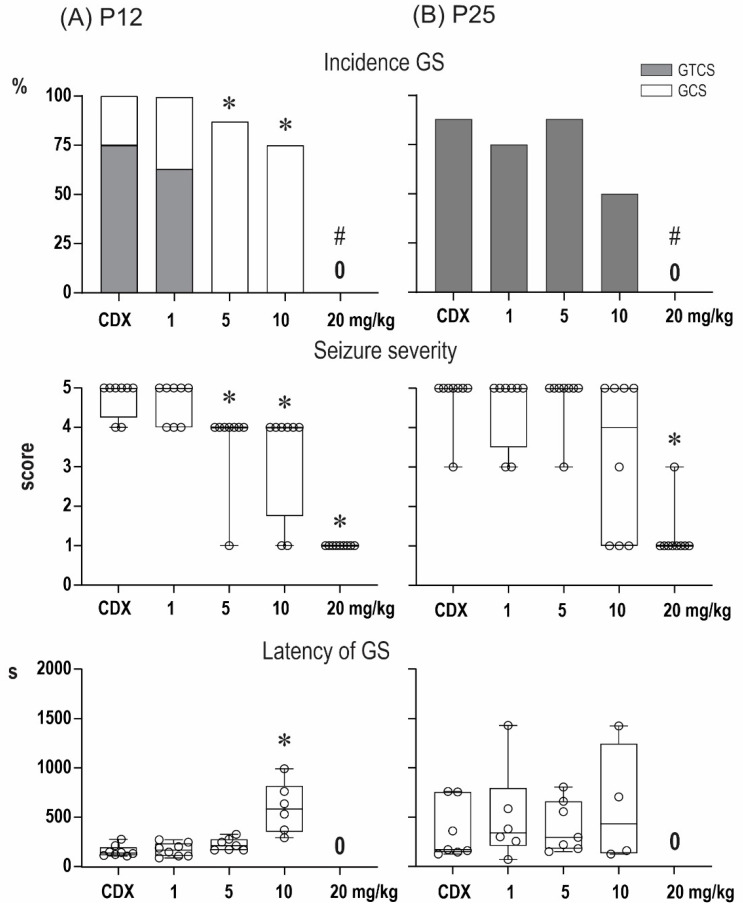
Effect of pregnanolone glutamate (PA-G) on seizures induced by pentylenetetrazol in 12- (**A**) and 25-day-old rats (**B**). From top to bottom: the incidence of generalized seizures (GS) is shown in columns (the black parts indicate complete generalized tonic-clonic seizures; the white parts show generalized clonic seizures without the tonic phase). The severity of seizures is expressed as a score [20], and the latencies of generalized seizures are shown as box plots (min to max) with individual values (circles). # denotes a significant difference in the incidence of GS compared with the controls, * denotes a significant difference in the incidence of TF in seizure severity and GS latency compared with the controls, and 0 means that none of the rats in the group exhibited GS.

The anticonvulsant effect of the compounds PA-S and PA-G are summarized in Figure 2 and Figure 3, respectively. In P12 animals, the administration of pentylenetetrazol (PTZ) at a dose of 100 mg/kg resulted in the development of generalized seizures (GS) in all vehicle-treated animals, and complete generalized tonic-clonic seizures (GTCS) with a tonic phase (TP) were observed in six out of eight animals. The two remaining animals developed generalized clonic seizures (GCS) without TP. In line with our previous studies, minimal, predominantly clonic, seizures with preserved righting reflexes (score 3) were not observed in P12 controls. As revealed by the Kruskal–Wallis test, PA-S at doses of 5 mg/kg and higher resulted in a significant decrease in seizure severity (H = 29.82; *p* < 0.0001). A decrease in seizure severity was driven mostly by specific suppression of the TP of GTCS (χ^2^ test (21.49, df = 4; *p* = 0.0002), and doses of 5 and 10 mg/kg completely abolished TP (Fisher’s test, *p* = 0.0015). In addition to the specific suppression of TP, PA-S tended to suppress the incidence of GS (χ^2^ test (22.16, df = 1; *p* < 0.0001), and a dose of 20 mg/mg completely eliminated GS in this age group (Fisher’s test *p* = 0.0003). As revealed by the Kruskal–Wallis test, the administration of PA-S at doses of 5 and 10 mg/kg significantly prolonged the latencies of GS (H = 13.08; *p* = 0.0045).

Pretreatment with PA-G suppressed seizure severity in the group of P12 animals (Kruskal–Wallis, H = 29.82; *p* < 0.0001). PAG, similar to PA-S, also decreased the incidence of TP (χ^2^ test (23.81, df = 4; *p* < 0.0001). In particular, doses of 5 and 10 mg/kg completely abolished TP (Fisher’s test *p* = 0.007). At the highest dose tested, PA-G prevented the development of GS in all animals (Fisher’s *p* = 0.0023). As revealed by the Kruskal–Wallis test, pretreatment with PA-G resulted in an increase in the latencies of GS (H = 16.42, *p* = 0.0009).

In vehicle-treated P25 animals, GTCS were observed in seven out of eight animals, i.e., all animals that developed GS exhibited TP. In contrast to P12 animals, minimal seizures (mS) are regularly observed in this age group, and this seizure type developed in five out of eight of the vehicle-treated rats. Pretreatment with PA-G decreased the seizure severity (Kruskal–Wallis test, H = 13.83, *p* = 0.0078), and post-hoc multiple comparisons revealed a lower score in animals pre-treated with 20 mg/kg of PA-S. In contrast to younger animals, PA-S did not exhibit specific effects against the TF of GTCS in P25 animals, but it affected the incidence of GS (χ^2^ test (13.50, 4; *p* = 0.009). Fisher’s tests, however, did not reveal any differences between individual treatment groups. Pre-treatment with PA-S did not affect any parameters of mS.

PA-G pre-treatment affected seizure severity (Kruskal–Wallis test, H = 24.19; *p* < 0.0001), and a dose of 20 mg/kg resulted in a decreased score (q = 0.0002). The effects on the incidence of GTCS (χ^2^ test (19.37, df = 4; *p* = 0.0007) were driven by a dose of 20 mg/kg, which completely suppressed these seizures (Fisher’s test, *p* = 0.0004). The latencies of GS were prolonged only by the 10 mg/kg dose of PA-G. Pre-treatment with PA-S did not affect any parameters of mS.

**Figure 4:**
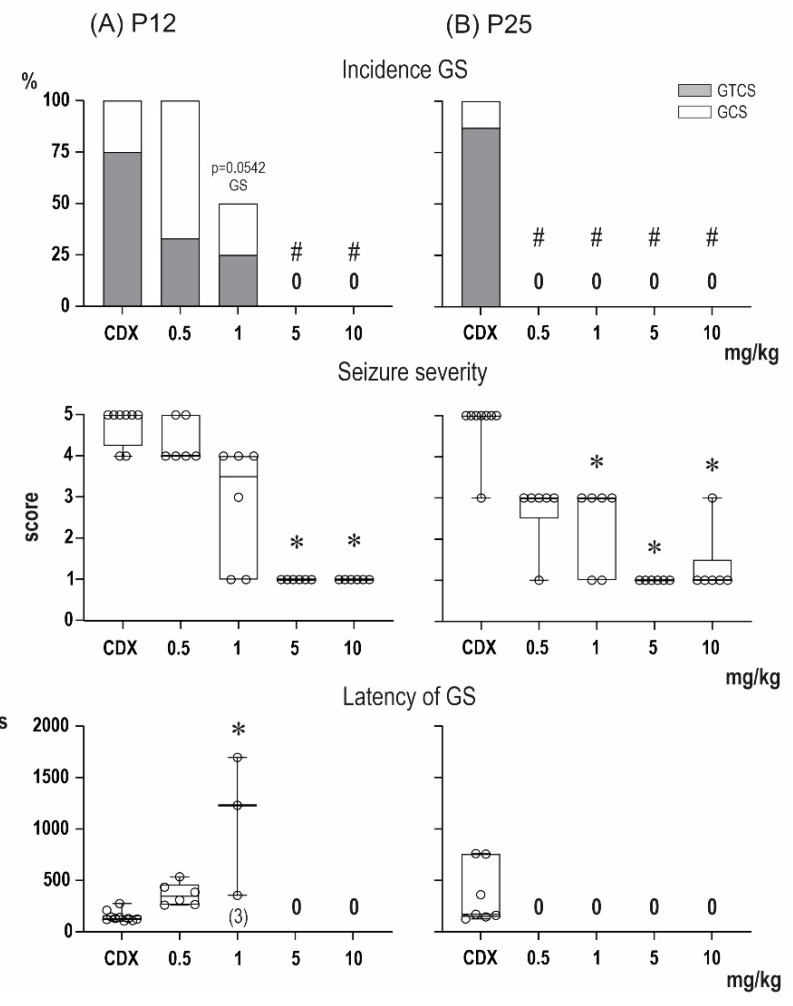
Effect of zuranolone on seizures induced by pentylenetetrazol in 12- (**A**) and 25-day-old rats (**B**). From top to bottom: the incidence of generalized seizures (GS) is shown in columns (the black parts indicate complete generalized tonic-clonic seizures; the white parts show generalized clonic seizures without the tonic phase). The severity of seizures is expressed as a score [20], and the latencies of generalized seizures are shown as box plots (min to max) with individual values (circles). # denotes a significant difference in the incidence of GS compared with the controls, * denotes a significant difference in the incidence of TF in seizure severity and GS latency compared with the controls, and 0 means that none of the rats in the group exhibited GS.

As our in vitro experiments demonstrated the ability of PA-G to modulate GABA_A_Rs in the micromolar range [21,22], we compared its anticonvulsant effect with the known potent GABA_A_Rs modulator zuranolone [23]. Zuranolone is an investigational NAS that acts as a positive allosteric modulator of GABA_A_Rs with an IC_50_ value of 7 nM for the allosteric displacement of [^35^S]-TBPS from the picrotoxin binding site on GABA_A_Rs [23]. Interestingly, the effects of zuranolone against PTZ-induced seizures differed both qualitatively and quantitatively from those of PA-G and PA-S (Figure 4). In P12 animals, the administration of zuranolone at doses of 5 and 10 mg/kg resulted in a significant decrease in seizure severity (Kruskal–Wallis test, H = 25.93; *p* < 0.0001). In contrast to the preferential effects of PA-G and PA-S against the tonic phase of GTCS, zuranolone suppressed the development of GS (χ^2^ = 25.98, df = 4; *p* < 0.0001), and GS were abolished by doses of 5 and 10 mg/kg in this age group. In contrast to PA-G and PA-S, zuranolone was more effective in P25 compared with P12 animals. As revealed by the Kruskal-Wallis test, zuranolone significantly decreased seizure severity (H = 26.88; *p* < 0.0001) at a dose of only 1 mg/kg and completely suppressed development of GS at all tested doses (χ^2^ = 25.98, df = 4; *p* < 0.0001). In addition, doses of 5 and 10 mg/kg suppressed the development of mS (χ^2^ = 16.44, df = 4; *p* = 0.0025). As revealed by the Kruskal–Wallis test, pretreatment with zuranolone resulted in the increase in the latencies of mS (H = 1.15, *p* = 0.003).

### 2.2. Steroidomic Analysis

**Figure 5:**
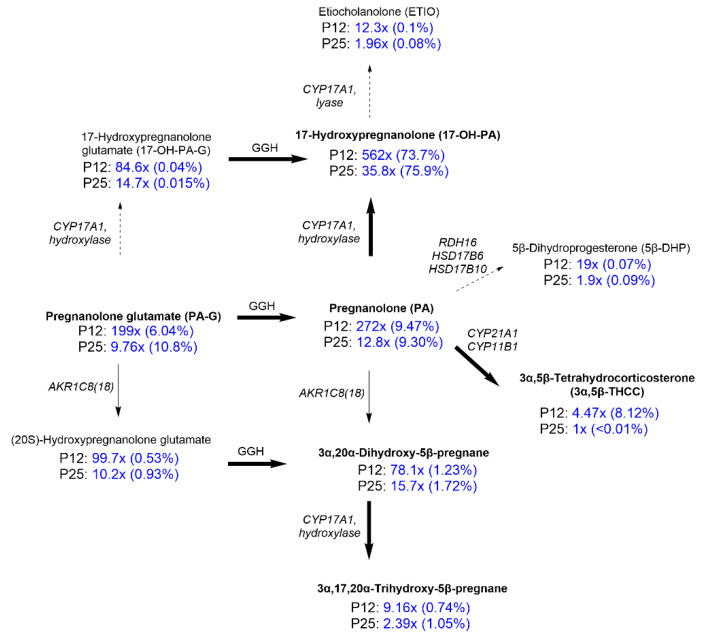
Steroidal metabolites identified in the hippocampus of male Wistar rats (P12 and P25 days old) after the administration of PA-G (i.p., 1 mg/kg in CDX). Only the steroids with significant increases above the basal levels (before PA-G application) are shown. The bold solid, thin solid, and thin dashed arrows indicate high, medium, and low conversion, respectively. The values below the name of the steroid compound represent the fold increase in the concentration of that particular compound and the percentage of total significantly increased steroids (in parenthesis) in the rat hippocampus above the basal levels 20 min after PA-G application. The names of steroids exceeding 1% of the total significantly increased 5β steroids are highlighted in bold. The abbreviations in italics represent steroidogenic enzymes in rats. AKR1C8(18): Type 8(18), subfamily 1C aldo-ketoreductase, CYP11B1: steroid 11β-hydroxylase; CYP17A1: steroid 17-hydroxylase-C17,20-lyase; CYP21A1: steroid 21-hydroxylase; GGH: γ-glutamyl hydrolase; HSD17B6: steroid type 6 17β-hydroxysteroid dehydrogenase; RDH16: Type 16 retinol dehydrogenase.

The hippocampus samples for metabolomic analysis of PA-G were obtained from male albino Wistar rats that were divided into six experimental groups consisting of 12- and 25-day-old animals (P12 and P25): a group of intact animals (I), control animals (C, i.p. application of a solution of 2-hydroxypropyl)-β-cyclodextrin (CDX)), and an experimental group (i.p. application of PA-G in a solution of CDX at a dose of 1 mg/kg). The results are summarized in Figure 5 (for details, see Supplementary Materials Table S1). The IUPAC chemical names and the commonly used trivial names of steroids are summarized in Table S2 (Supplementary Materials).

Our data show (Figure 5) that the majority of PA-G was metabolized to unconjugated 17-hydroxypregnanolone glutamate (17-OH-PA). At the same time, we did not detect measurable concentrations of either Δ^5^-steroid (17α-hydroxypregn-5-en-20-one) or Δ^4^-steroid (17α-hydroxyprogesterone) in the rat brain. We also found only a low concentration of etiocholanolone (ETIO, 0.1% and 0.08%) of which the biosynthesis could be catalyzed by CYP17A1. A moderate quantity of 3α,5β-tetrahydrocorticosterone (3α,5β-THCC) was measured in the hippocampi of younger pups after PA-G administration. This amount is approximately 8% of the total 5β steroids; in the older pups (Day 25), it represented less than 0.01%. The concentration of PA-G found in the hippocampi represents approximately 6% to 10% of the brain’s 5β-steroids in the hippocampus after PA-G administration. Besides the 20-oxo-5β-pregnanes, we observed pronouncedly increased levels of their 20α-dihydro-metabolites; however, their quantities represented only 0.74–1.72% of the total 5β-steroids. A minor quantity of a 3-oxo metabolite of PA, namely 5β-dihydroprogesterone, after PA-G administration was also detectable.

Concerning the effect of PA-G administration (i.p., 1 mg/kg) on the levels of naturally occurring NS (Table 1, Figure 1A,C), our results demonstrated that in intact animals, the brain concentrations of pregnenolone (PE), pregnenolone sulfate (PE-S), dehydroepiandrosterone (DHEA), dehydroepiandrosterone sulfate (DHEA-S), androstenediol, androstenediol sulfate, PA, 3α,20α-dihydroxy-5β-pregnane, 17-OH-PA, 3α,17α,20α-trihydroxy-5β-pregnane, and ETIO were physiologically higher in 12- than in 25-day-old rats.

Next, our study demonstrated that the PA-G-treated animals have significantly increased concentrations of compounds that are metabolites of PA-G, particularly the compounds PA, conjugated PA, 3α,20α-dihydroxy-5β-pregnane, conjugated 3α,20α-dihydroxy-5β-pregnane, 17-OH-PA, conjugated 17-OH-PA, 3α,17α,20α-trihydroxy-5β-pregnane, and ETIO. It is important to highlight that the absolute concentrations in P12 animals increased by more than a hundredfold, i.e., the concentration of PA increased by up to 272-fold and the concentration of 17-OH-PA increased by up to 560-fold. A similar trend of increased levels of PA and 17-OH-PA was measured for P25 animals, but with a lower fold increase. The concentration of PA increased by 13-fold and 36-fold for 17-OH-PA.

As regards the compounds that are not metabolites of PA-G, the results showed direct modulation of NS levels after PA-G administration. Namely, the compound PE displayed significantly decreased concentrations in PA-G-treated animals compared with the controls.

In contrast, the compound DHEA-S displayed significantly increased concentrations in PAG-treated animals compared with the controls. Compound PE-S showed a trend similar to DHEA-S, although the results were not statistically significant. Brain concentrations of DHEA, ALLO, androstenediol, and androstenediol sulfate did not seem to be affected.

## 3. Discussion

In the current article, we studied the anticonvulsant effect of the NAS pregnanolone glutamate (PA-G) [24,25] in a model of pentetrazol-induced seizures in 12- and 25-day-old animals. As a comparison, endogenous NS, pregnanolone sulfate (PA-S), and the neuroactive steroid zuranolone were used [16]. The expected neuroprotective effect was based on the combined in vitro effects of GABA_A_R agonism and NMDAR antagonism. This concept of the neuroprotective effect of NAS that inhibits NMDAR while also potentiating GABA_A_R function has been recently proposed for the treatment of neuropsychiatric disorders [17].

The compound PA-S inhibits NMDA-induced currents in transfected HEK293 cells with an IC_50_ value of 24 µM [26] and acts as a negative modulator of GABA_A_Rs at micromolar concentrations [13]. PA-G is a synthetic analog of PA-S with the ability to inhibit NMDA-induced currents in transfected HEK293 cells with an IC_50_ value of 73 µM [25]. In terms of the GABAergic activity, PA-G was found to increase the I_GABA_ peak in a dose-dependent manner, with an EC_50_ value of 7 µM, and to increase the I_GABA_ peak amplitude with a maximum potentiation of up to 422% in isolated pyramidal neurons of the rat hippocampus [22]. Finally, we demonstrated the neuroprotective effect of PA-G in several biological models in vivo, [24,27], including a model of ischemia in immature rats [28].

Our experiments on the anticonvulsant effects in vivo demonstrated that pre-treatment with PA-G or PA-S in a model of epileptic seizures elicited by pentylenetetrazole afforded selective suppression of the tonic phase of generalized seizures in 12-day-old rats. In particular, PA-G pre-treatment of P12 animals at doses of 5 and 10 mg/kg completely abolished the tonic phase, and at a dose of 20 mg/kg, PA-G pre-treatment prevented the development of generalized seizures in all animals. Finally, pretreatment with PA-G resulted in an increase of the latencies of generalized seizures in P12 animals. In contrast, selective suppression of the tonic phase was not observed in the P25 animals, and P25 animals that were pre-treated with PA-G/PA-S exhibited only a moderate effect on the incidence of generalized seizures, reaching the level of statistical significance only after the 20 mg/kg dose of PA-G. It can be therefore concluded that the effects of PA-S and PA-G are age-specific.

The phenomenon of the age specificity of neuroprotection could be related to the fact that endogenous NS are synthesized abundantly in the perinatal brain while playing an important role in CNS development and self-protection. For example, pregnancy is characterized by extremely elevated concentrations of most neurosteroids in both free and conjugated form in the maternal and fetal brain [29,30,31,32]. NS are produced during pregnancy by the fetal adrenal glands and the placenta in synergy with the maternal and fetal livers [33]. Their function is still not fully understood, but they apparently contribute to the stabilization of pregnancy and participate in the neurohumoral stress response of women during childbirth. In particular, NS are hypothesized to have a protective function against ischemic–hypoxic damage in the perinatal period when the fetus can increase NS production in response to protentional injury as prevention against cognitive or motor damage [31,34]. In connection with the topic of fetal neuroprotection, the most mentioned neurosteroids are the positive modulators of GABA_A_R, allopregnanolone and pregnanolone [31,32,35,36].

Childhood epilepsy is epilepsy of a special kind. An immature brain generates epileptic seizures more easily than an adult brain. The reason for this phenomenon is the need for a more excitatory brain in the early stages of development for learning. Epileptic encephalopathies occur in the earliest age categories (newborns, infants, and toddlers) [37,38]. In these syndromes, the epileptic activity causes developmental arrest and, in some cases, developmental regression. The term “catastrophic epilepsy” has been given to these syndromes because in about half the cases, epilepsy stops the progress of normal brain development in affected children [39].

Considering the in vitro activity of PA-G on GABA_A_Rs and NMDARs in the micromolar range [21,22,25], we compared PA-G’s effect with a potent positive allosteric modulator of GABA_A_Rs, zuranolone, a novel neurosteroid developed recently by Sage Therapeutics [23]. In a Phase 3 clinical trial, zuranolone improved the symptoms of depression in women with postpartum depression [40]. Zuranolone is also being further developed for the treatment of major depressive disorders [41]. On the other hand, no studies describing its anticonvulsant effects have been published. Such results are of great interest, as zuranolone is a potent modulator of both synaptic and extrasynaptic receptor subtypes in the subnanomolar range [23]. Therefore, we evaluated its anticonvulsant effects in a model of PTZ-induced seizures in groups of both P12 and P25 animals. As expected, the effective dose for a neuroprotective effect was significantly decreased compared with PA-G. Interestingly, our results also demonstrated that the effects of zuranolone differed both qualitatively and quantitatively from those of PA-G and PA-S. In P12 animals, the administration of zuranolone at doses of 5 and 10 mg/kg resulted in a significant decrease in seizure severity. In contrast to the preferential effects of PA-G and PA-S against the tonic phase of GTCS, zuranolone suppressed the development of GS that were abolished by doses of 5 and 10 mg/kg in this age group. In contrast to PA-G and PA-S, zuranolone was more effective in P25 compared with P12 animals.

The aforementioned results indicate that the in vivo anticonvulsant effects of PA-G/PA-S may not be that closely related to GABA_A_Rs and NMDARs. This conclusion is supported by the anticipated metabolic instability. In particular, for PA-G, the ester bond connecting the glutamate moiety to the steroid skeleton could be susceptible to plasmatic degradation by esterases. Therefore, we performed a metabolomic study of rat hippocampus samples in P12 vs. P25 animals that were obtained after the administration of PA-G. Our results demonstrated that 20 min after PA-G administration, only approximately 6–10% of the PA-G remained in the original form and approximately 10% in the form of its unconjugated metabolite, PA. As the major metabolite, the compound 17-OH-PA was identified. The concentrations of 17-OH-PA increased by up to 560-fold in P12 animals compared with basal levels. In P25 animals, the concentration of 17-OH-PA increased by 36-fold. Similar trends were observed for the minor metabolite PA. The concentrations of PA increased by up to 272-fold in P12 animals and by up to 13-fold in P25 animals. It is important to mention that the distinct permeability of the blood–brain barrier between P12 and P25 animals affects this age specificity.

According to our study, the majority of administered PA-G is converted to 17-OH-PA. Unfortunately, the current literature describing the biological effect of 17-OH-PA is very limited. Several studies described 17-OH-PA as a potential biological marker for the diagnosis of congenital adrenal hyperplasia, [42] a genetic disorder that affects the adrenal glands that is caused by a 21-hydroxylase deficiency [43,44,45]. The only study relevant to our research described the subtype-selective effects of various neurosteroids on human α4β3δ and α4β3γ2 GABA_A_Rs [46]. The α4β3δ GABA_A_Rs are extrasynaptic receptors, while α4β3γ2 GABA_A_Rs represent an intermediary receptor between the previously mentioned extrasynaptic and synaptic α1β3γ2 GABA_A_Rs. The compound 17-OH-PA was identified as a weak modulator of the α4β3γ2 receptor, as the greatest response of 20% occurred at 100 μM. At the α4β3δ receptors, 17-OH-PA was able to potentiate the responses with an EC_50_ value of 3.5 µM with a maximum efficacy of 57%.

Taken together, our results from in vivo studies, supported by the published pharmacokinetic data of PA-G [24], indicate that compound PA-G may act as a pro-drug of 17-OH-PA. The mechanism of 17-OH-PA is not known, and further detailed studies should be performed to evaluate its in vitro and in vivo effects. The synthesis of neuroactive 17-OH-PA’s conjugates should be also considered as well.

After that, we measured the levels of neurosteroids following the acute administration of PA-G. We hypothesized that the metabolome of NS induced by the external application of synthetic NAS could play a significant role. To the best of our knowledge, no complex study describing the level of NS after the administration of a synthetic NAS has been published. These neurochemical changes can lead to a strong anticonvulsant and neuroprotective effect. Indeed, recent studies showed a correlation between reduced levels of neurosteroids and their potential therapeutic effect in various models of seizures. Strong evidence that levels of NS are decreased in both animals and patients with epilepsy suggest a diminished regulatory function of GABA_A_Rs in the CNS [11,47,48]. Consequently, treatment with neurosteroids modulating GABA_A_Rs, e.g., ALLO or PA, has served as a compensatory mechanism [48,49,50].

In addition, we described the effect of PA-G administration (i.p., 1 mg/kg) on the levels of naturally occurring NS. Our results demonstrated that in intact animals, the brain concentrations of PE, PE-S, DHEA, DHEA-S, androstenediol, androstenediol sulfate, PA, 3α,20α-dihydroxy-5β-pregnane, 17-OH-PA, 3α,17α,20α-trihydroxy-5β-pregnane, and ETIO were physiologically higher in 12- than in 25-day-old rats. As expected, our study demonstrated that the PA-G-treated animals had significantly increased concentrations of compounds that are metabolites of PA-G. Surprisingly, our study showed the direct modulation of NS levels that are not metabolites of PA-G. Specifically, the compound PE displays significantly decreased concentrations in PA-G-treated animals compared with the controls. The reason for this phenomenon remains unclear. We hypothesize that the administration of PA-G, which is followed by its chemical transformation into 17-OH-PA, may serve as a compensatory mechanism of the stress reaction and that therefore, the synthesis of corticosteroids via their precursor PE is diminished. In contrast, the compound 17-oxo-androst-5-en-3β-yl 3-sulfate (DHEA-S) displayed significantly increased concentrations in PA-G-treated animals compared with the controls. This trend that was observed for DHEA-S was also observed for PE-S, although it was not statistically significant. These metabolomic results showed that in P12 animals, the physiological concentrations increased. Administration of the synthetic neuroprotective PA-G affected the levels of NS.

Concerning the steroidomic aspects, our results indicated that the majority of PA-G metabolized to 17-OH-PA after facile hydrolysis of a glutamate ester group at position C-3 by a γ-glutamyl hydrolase enzyme (GGH). GGH is most active in the small intestine, and represents a major metabolic pathway in extra-adrenal peripheral tissues [51]. After PA-G is metabolized into 17-OH-PA, it penetrates across the blood–brain barrier (BBB) into the CNS. In our experiments, 17-OH-PA represented about three-quarters of the brain’s 5β-steroids in the tissue 20 min after PA-G administration. These results demonstrate that the activity of the steroid C17-hydroxylase-C17,20-lyase (CYP17A1) in the CNS is marginal compared with the activity of GGH [52,53,54,55,56,57,58,59]. In other words, the participation of the brain’s CYP17A1 activity in forming 17-OH-PA after PA-G administration is negligible. Although rodent adrenals do not express the CYP17A1 gene [59], these data point to an intensive extra-adrenal 17-hydroxylation of a steroidal pregnane skeleton in the peripheral nervous system before their penetration into the CNS.

The very low concentrations of ETIO (0.1% and 0.08%) in the analyzed tissue (Figure 5) imply that the lyase activity of the CYP17A1 enzyme is minor, despite an approximate 12- and 2-fold increase in the ETIO concentration for P12 and P25 animals, respectively. The aforementioned finding unambiguously demonstrates the functioning of the so-called “backdoor” pathway of the 5β-steroids in the rats. The PA is directly converted to 17-OH-PA and ETIO in the sequence, apart from the classical “front door” pathway that is based on CYP17A1-catalyzed conversion of the C21,Δ^5^ and C21,Δ^4^ steroids to their 17-hydroxy- and C19- metabolites in the sequence, followed by a subsequent reduction in the latter substances in the C-5 and C-3 positions [60]. Note, however, that the “front door” pathway cannot be associated with PA-G administration, as the metabolic pathway from PE to PA is irreversible.

Our data also indicate the direct peripheral conversion of PA to 3α,5β-THCC, which is sequentially catalyzed by the steroid 21-hydroxylase (CYP21A1) and the steroid Type 1 11β-hydroxylase (CYP11B1), apart from the corticosterone formation and its subsequent reductive catabolism. Afterwards, 3α,5β-THCC penetrates through the BBB into the CNS. This passive transport, however, functions in the younger pups (Day 12) only, as the percentage of 3α,5β-THCC in the hippocampi of the younger pups after PA-G administration was approximately 8% of the total 5β-steroids, while in the older pups (Day 25), it represented less than 0.01%. This was almost certainly a result of the less penetrable BBB in the latter group. Moreover, the group of the older animals had consistently lower values of the increase in 5β-steroids, which showed significant changes after PA-G administration.

Some previous data, including our own studies, demonstrated a close association between the circulating steroids and steroids in the brain [61] and cerebrospinal fluid [62]. Our present data demonstrate, in agreement with the previously mentioned studies, that a substantial concentration of PA-G directly penetrates through the BBB, representing approximately 6% to 10% of the brain’s 5β-steroids in the hippocampus after PA-G administration. As expected, the proportion was lower for the more polar 17-OH-PA-G, as the steroid’s penetrability through the blood–brain barrier negatively correlates with the polarity of the substance (reviewed in our previous study [62]).

The penetrability of steroid conjugates (i.e., sulfates or glutamates) from the peripheral nervous system into the CNS can be significantly affected by the nature of the C-3 substituent or the affinity of steroids towards membrane transporters. For example, PE-S and, to a less extent, the more polar DHEA-S (17-oxo-androst-5-en-3β-yl 3-sulfate) readily penetrate through the BBB, despite the known effect of organic anion transporters preferring about 10 times the efflux of steroid sulfates from the CNS over their influx [63]. Both conjugates (PE-S and DHEA-S) underwent a consequent extensive (>50%) desulfation within 0.5 min of uptake [63]. While a small portion of DHEA-S after desulfation was slowly metabolized (within 24 h), no signs of such catabolism were found in the PE-S. Furthermore, an earlier study by Wang et al. [64] also demonstrated the transport of peripherally applied PE-S across the BBB. Its penetration across the BBB into the brain was about 10 times slower when compared with its unconjugated counterpart. In all probability, the penetration of the less polar steroidal C-3-glutamates should be even easier when compared with the more polar steroidal C-3-sulfates.

Furthermore, our data demonstrated that both PA-G and unconjugated PA are readily converted into their 20α-dihydroxy metabolites by Type 8(18), subfamily 1C aldo-ketoreductase (AKR1C8(18)) [65]. However, this conversion was less intense than the 17-hydroxylation. The 20α-HSD activity transforming 20-oxo-pregnanes to their 20α-hydroxy counterparts has been reported, even in the rat hypothalamus [66].

Besides the aforementioned metabolic steps, a minor conversion of PA to 5β-dihydroprogesterone (5β-DHP) was also apparent. This oxidative metabolic step converting 3α/β- reduced steroids to their 3-oxo counterparts may be catalyzed by several enzymes such as Type 16 retinol dehydrogenase (RDH16), the steroid Type 6 17β-hydroxysteroid dehydrogenase (HSD17B6), and/or the steroid Type 10 17β-hydroxysteroid dehydrogenase (HSD17B10).

Finally, we aimed to compare the absolute concentrations of NS (ng/g) after PA-G administration with the physiological concentrations measured by other authors. Unfortunately, the literature available is very limited. Most of the studies are rather technical, describing method development and validation, sample derivatization, steroid extraction and separation, etc. Furthermore, we are not aware of a metabolomic study describing the levels of NS in P12 and P25 animals compared with adult animals (P60). According to the literature, brain concentrations measured for PE vary from 0.6 to 9 ng/g [67,68,69,70,71,72], the concentrations of PE-S vary from 8 to 14 ng/g [68,69], the concentrations of DHEA vary from 0.1 to 1.7 ng/g [68,69,70,72], the concentration of DHEA-S was measured as 2.5 ng/g [68], the concentrations of PA vary from 0.1 to 3 ng/g [70,73], and the concentrations of ALLO vary from 0.3 to 0.4 ng/g [70,71,72]. If we take these findings together, the measurement of physiological NS levels is method-dependent.

The identification of endogenous 17-OH-PA as a potential neuroprotective compound that is able to modulate the levels of endogenous NS is the main outcome of the present study, which offers new avenues for further investigation. For example, the published studies have highlighted the promise of NS replacement therapy in catamenial epilepsy, a condition that exhibits an incidence of 40% among women [74]. Whether there is a link between the difference in NS levels and the neuroprotective effect remains to be further investigated.

## 4. Materials and Methods

### 4.1. Animals

Experiments were performed in male Wistar albino rats (total *n* = 258, Institute of Physiology of the Czech Academy of Sciences) on two postnatal days (P12 and 25). The day of birth was defined as Day 0, and animals were weaned at P21. Rats were housed in a controlled environment (temperature 22 ± 1 °C, humidity 50–60%, lights on from 6 a.m. to 6 p.m.) with free access to food and water. During the experiments with pups, the temperature in plexiglass cages was maintained at 32 ± 2 °C using an electric heating pad connected to a digital thermometer to compensate for the immature thermoregulatory function at this age [75].

All procedures involving animals and their care were conducted according to the ARRIVE guidelines https://www.nc3rs.org.uk/arrive-guidelines (access date 28 November 2021) in compliance with national (Act No. 246/1992 Coll.) and international laws and policies (EU Directive 2010/63/EU for animal experiments and the National Institutes of Health Guide for the Care and Use of Laboratory Animals, NIH Publication No. 8023, revised 1978). The experimental protocol was approved by the Ethical Committee of the Czech Academy of Sciences (Approval No. 15/2018).

### 4.2. Pregnanolone Glutamate, Pregnanolone Sulfate, and Zuranolone

The compounds pregnanolone glutamate (20-oxo-5β-pregnan-3α-yl L-glutamyl 1-ester, PA-G) and pregnanolone sulfate (pyridinium 20-oxo-5β-pregnan-3α-yl sulfate, PA-S) were synthesized according to the literature [25,26]. The compound zuranolone is commercially available (ChemShuttle, Hayward, CA, USA, catalog number 186443, CAS 1632051-40-1). PA-G, PA-S, and zuranolone were dissolved in a solution of 3 g of (2-hydroxypropyl)-β-cyclodextrin (CDX, Sigma-Aldrich, St. Louis, MO, USA) and 157 mg of citric acid (Sigma-Aldrich, St. Louis, MO, USA) in 30 mL of distilled water. The pH was adjusted to 7.36 with NaOH (Sigma-Aldrich, St. Louis, MO, USA).

### 4.3. PTZ-Induced Seizures

Solutions of PA-G and PA-S were administered intraperitoneally at a concentration of 1 mg/mL, and doses of 1, 5, 10, and 20 mg/kg were used. The solutions of zuranolone were administered intraperitoneally at a concentration of 1 mg/mL, and doses of 0.5, 1, 5, and 10 mg/kg were used. Controls were injected with CDX solution in a volume corresponding to the highest dose (i.e., 20 mL/kg). The same control group was used for PA-S and PA-G. Pentylenetetrazol (PTZ, Sigma Aldrich, Gillingham, Dorset, UK) was dissolved in water in a concentration of 50 mg/mL and was administered subcutaneously at a dose of 100 mg/kg 20 min after the administration of PA-G or PA-S. Each age and dose group comprised 8 animals. The rats were placed individually into plexiglass boxes and were then observed for 30 min after PTZ injection. The incidence, severity, and latencies of seizures were assessed. Younger animals were placed on a pad heated to 34 °C, i.e., to the temperature in the nest for the whole duration of the experiment because of immature thermoregulation [75]. Generalized tonic-clonic or clonic seizures with a loss of righting reflexes were present after a dose of 100 mg/kg of PTZ in all control animals in both age groups. As the first seizure, older animals might exhibit the so-called minimal pentetrazol seizures, i.e., clonic seizures of the head and forelimb muscles with preserved righting ability. The severity of seizures was quantified using a five-point scale [20] as follows: 0—no changes; 0.5—abnormal behaviour (e.g., automatisms, increased orienting reaction); 1—isolated myoclonic jerks; 2—atypical or prolonged minimal seizures; 3—clonic seizures (mS) involving head and forelimb muscles with preserved righting reflexes (older-term minimal pentetrazol seizures); 4—generalized seizures without the tonic phase (GCS); 5—complete generalized tonic-clonic seizures (GTCS).

### 4.4. Brain Samples for Metabolomic Analysis

In a separate experiment, samples of the hippocampus were obtained from male albino Wistar rats in 6 experimental groups consisting of P12 and P25 animals that were either intact (I), controls (C, i.p. application of a solution of CDX), or in the experimental group (i.p. application of PA-G in a solution of CDX at a dose of 1 mg/kg). Each group consisted of 5 animals. The animals were sacrificed using ether anaesthesia (Penta, Czech Republic) 30 min after administration of the drug. The hippocampi were quickly removed, weighed, and frozen at −80 °C in sterile plastic microvials until analysis.

### 4.5. Chemical Analysis

Steroidal and deuterated standards were purchased from Steraloids (Newport, RI, USA). The deuterated standard D7 cortisone [2,2,4,6,6,12,12-D7] and trimethylchlorosilane (TMCS) for the hydrolysis of steroids conjugates were from Sigma-Aldrich (St. Louis, MO, USA). Sylon BTZ, methoxyamine hydrochloride, and all other solvents and chemicals were from Merck (Darmstadt, Germany). All solvents were of HPLC grade.

### 4.6. Sample Preparation

An amount of 17–100 mg of the hippocampal tissue was minced and then administered into screw-cap tubes. Next, 2 mL of methanol with a mixture of internal standards was added, and the samples were incubated for 1 week in a refrigerator at 4 °C with agitation for 2 min once a day. Afterwards, the extract was transferred into new screw-cap tubes and dried in a vacuum centrifuge at 45 °C. After that, 1 mL of water of chromatographic purity was added, and the samples were further treated as described in our recently published paper [76]. The GC-MS/MS-based steroid quantification method was described in detail in our recent article [76].

The process of separating conjugated and unconjugated steroids has been described in detail in the literature [76]. Briefly, the unconjugated steroids were extracted from a testing sample with diethyl-ether, the extract was dried, and the lipids were separated by partitioning between a mixture of methanol with water (4:1, *v/v*) and pentane. The pentane phase was discarded, and the polar phase was dried and used for further analysis of the unconjugated steroids. Steroid conjugates remaining in the polar residue after diethyl ether were hydrolyzed to their parent hydroxy steroids (unconjugated molecules) and analyzed.

### 4.7. Nomenclature of Endogenous Steroids and Metabolites of PA-G

The IUPAC chemical names, commonly used trivial names, and abbreviations of the steroids to for this study are summarized in Table S2 (Supplementary Materials).

### 4.8. Statistics

The sample size was determined in advance according to previous experience with the given models and followed the principles of the three Rs (Replacement, Reduction, and Refinement; https://www.nc3rs.org.uk/the-3rs, access date 28 November 2021). Outcome measures and statistical tests were prospectively selected. At the beginning of the study, simple randomization was used to assign each animal to a particular treatment group. Data acquisition and analysis were carried out by researchers blinded to the treatment. Data were analyzed using GraphPad Prism 8 (GraphPad Software, San Diego, CA, USA) software. Using the D’Agostino-Pearson normality test, all datasets were first analyzed to determine whether the values were derived from a Gaussian distribution. Differences in anticonvulsant activity between controls and neurosteroid-treated animals were analyzed using ordinary one-way ANOVA with post-hoc multiple comparison by controlling the false discovery rate (FDR). The incidence of individual seizure phenomena was compared first with an χ^2^-test for trends, and subsequently in control and individual dose groups using Fisher’s exact test. For interpretation of the results, the calculated value of χ^2^ was compared with the values in the χ^2^ distribution table (https://www.statology.org/chi-square-distribution-table/, 28 November 2021) and a *p*-value < 0.05 was required for significance and q < 0.05 was taken as discovery.

## 5. Conclusions

The results of this study indicate that synthetic neurosteroids represent an attractive target for studying perinatal neural diseases in animal models due to their anticonvulsant and neuroprotective effects. In particular, we have described the synthetic compound pregnanolone glutamate (PA-G) in a model of pentylenetetrazol-induced seizure in 12-day-old and 25-day-old animals. The compound PA-G demonstrated a significantly pronounced effect in the early perinatal period (P12 animals). The compound 17-hydroxy-pregnanolone (17-OH-PA) was identified as the major metabolite. The mechanism of action of 17-OH-PA is unknown, and the literature available is very limited. Our findings suggest that PA-G may act as a pro-drug for endogenous 17-OH-PA. Finally, the effect of administering the synthetic compound PA-G on the level of endogenous neurosteroids was described, demonstrating the direct modulation of unexpected neurosteroid levels, namely pregnenolone and dehydroepiandrosterone sulfate. However, as regards the contribution of the above data to the study of human physiology and pathophysiology, account should also be taken of the differences between steroidogenesis in humans and rodents.

## Figures and Tables

**Table 1:** Steroid concentrations (ng/g or pg/g) in hippocampal tissue of rats (12- and 25-day-old animals) 20 min after PA-G administration (i.p., 1 mg/kg in CDX).

Steroid	Age (Days)	Control (C) ^1^	Intact (I) ^2^	PA-G-Treated (P) ^3^
Pregnenolone (PE) (ng/g)	12	11.3 (9.4, 13.5)	9.93 (8.21, 11.9)	4.69 (3.74, 5.8)
	25	5.68 (4.59, 6.97)	4.56 (3.39, 6)	3.44 (2.68, 4.34)
Age: F = 24.8, *p* < 0.001, Group: F = 12.8, *p* < 0.001, Age × Group: F = 1.9, *p* = 0.176; C25 < C12, I25 < I12, P < I, P < C, P12 < I12, P12 < C12				
Pregnenolone sulfate (PE-S) (ng/g)	12	16.9 (12.6, 22.6)	24 (17.3, 33.4)	29.5 (22, 39.9)
	25	8.81 (6.41, 11.9)	9.86 (7.22, 13.3)	10.5 (7.74, 14.2)
Age: F = 25.9, *p* < 0.001, Group: F = 1.7, *p* = 0.205, Age × Group: F = 0.5, *p* = 0.626; C25 < C12, I25 < I12, P25 < P12				
Dehydroepiandrosterone (DHEA) (ng/g)	12	1.01 (0.829, 1.26)	1.37 (1.09, 1.78)	1.13 (0.916, 1.42)
	25	0.779 (0.648, 0.946)	0.668 (0.56, 0.803)	0.644 (0.54, 0.773)
Age: F = 21.2, *p* < 0.001, Group: F = 0.3, *p* = 0.746, Age × Group: F = 1.4, *p* = 0.277; I25 < I12, P25 < P12				
Dehydroepiandrosterone sulfate (DHEA-S) (ng/g)	12	2.5 (2.05, 3.11)	3.68 (2.83, 5.02)	4.86 (3.69, 6.81)
	25	1.42 (1.19, 1.7)	1.3 (1.09, 1.55)	1.52 (1.27, 1.82)
Age: F = 63.2, *p* < 0.001, Group: F = 2.6, *p* = 0.097, Age × Group: F = 1.8, *p* = 0.187; C25 < C12, I25 < I12, P25 < P12, P12 > C12				
Androstenediol (ng/g)	12	0.57 (0.465, 0.709)	0.846 (0.67, 1.1)	0.758 (0.606, 0.968)
	25	0.39 (0.323, 0.474)	0.463 (0.373, 0.582)	0.364 (0.302, 0.441)
Age: F = 23.2, *p* < 0.001, Group: F = 1.8, *p* = 0.191, Age × Group: F = 0.7, *p* = 0.49; I25 < I12, P25 < P12				
Androstenediol sulfate (ng/g)	12	5.03 (4.47, 5.69)	7.18 (6.21, 8.34)	6.07 (5.36, 6.89)
	25	1.44 (1.28, 1.62)	1.39 (1.24, 1.57)	1.62 (1.44, 1.82)
Age: F = 459, *p* < 0.001, Group: F = 2.3, *p* = 0.127, Age × Group: F = 2.8, *p* = 0.085; C25 < C12, I25 < I12, P25 < P12, I12 > C12				
Allopregnanolone (ng/g)	12	0.139 (0.0834, 0.218)	0.254 (0.165, 0.378)	0.246 (0.159, 0.367)
	25	0.151 (0.0919, 0.235)	0.119 (0.0696, 0.19)	0.144 (0.0869, 0.224)
Age: F = 2.6, *p* = 0.118, Group: F = 0.4, *p* = 0.672, Age × Group: F = 1, *p* = 0.384				
5β-Dihydroprogesterone (pg/g)	12	25.2 (15.8, 39)	92.9 (54.8, 173)	477 (214, 1810)
	25	49.6 (30.4, 83.2)	48.8 (31.5, 77.2)	94.3 (58.6, 164)
Age: F = 1.7, *p* = 0.21, Group: F = 11.5, *p* < 0.001, Age × Group: F = 4.2, *p* = 0.029; P25 < P12				
Pregnanolone (ng/g)	12	0.234 (0.162, 0.338)	0.254 (0.183, 0.353)	63.6 (26, 200)
	25	0.393 (0.281, 0.553)	0.106 (0.0704, 0.156)	5.02 (3.01, 8.95)
Age: F = 6.9, *p* = 0.016, Group: F = 131.6, *p* < 0.001, Age × Group: F = 7.2, *p* = 0.004; I25 < I12, P25 < P12, P > I, P > C, P12 > I12, P12 > C12, I25 < C25, P25 > I25, P25 > C25				
Conjugated pregnanolone (ng/g)	12	0.204 (0.144, 0.285)	0.381 (0.265, 0.546)	40.6 (18.7, 110)
	25	0.615 (0.445, 0.859)	0.237 (0.162, 0.342)	6 (3.7, 10.4)
Age: F = 0.3, *p* = 0.581, Group: F = 115.8, *p* < 0.001, Age × Group: F = 11.5, *p* < 0.001; C25 > C12, P25 < P12, P > I, P > C, P12 > I12, P12 > C12, I25 < C25, P25 > I25, P25 > C25				
3α,20α-Dihydroxy-5β-pregnane (ng/g)	12	0.108 (0.0809, 0.144)	0.339 (0.248, 0.472)	8.42 (4.58, 17.2)
	25	0.0583 (0.0431, 0.078)	0.0284 (0.0186, 0.041)	0.919 (0.632, 1.38)
Age: F = 75.2, *p* < 0.001, Group: F = 129.5, *p* < 0.001, Age × Group: F = 9.5, *p* = 0.001; C25 < C12, I25 < I12, P25 < P12, P > I, P > C, I12 > C12, P12 > I12, P12 > C12, P25 > I25, P25 > C25				
Conjugated 3α,20α-dihydroxy-5β-pregnane (ng/g)	12	35.8 (22.6, 55.8)	180 (100, 335)	3570 (1820, 7580)
	25	50.6 (32.4, 78.5)	77.8 (50.2, 121)	518 (311, 899)
Age: F = 5.9, *p* = 0.024, Group: F = 53.7, *p* < 0.001, Age × Group: F = 4.3, *p* = 0.026; P25 < P12, I > C, P > I, P > C, I12 > C12, P12 > I12, P12 > C12, P25 > I25, P25 > C25				
17-Hydroxyallopregnanolone (ng/g)	12	7.18 (3.2, 14)	20 (11.6, 32.4)	8.36 (4.24, 14.9)
	25	14 (7.76, 23.5)	7.57 (3.76, 13.7)	3.19 (1.2, 6.63)
Age: F = 1.3, *p* = 0.266, Group: F = 2.2, *p* = 0.138, Age × Group: F = 2.4, *p* = 0.117				
Conjugated 17-hydroxyallopregnanolone (pg/g)	12	26.5 (13.8, 45.2)	21.8 (10.7, 38.5)	18.9 (7.92, 36.4)
	25	45.3 (24.7, 75.8)	40.9 (21.8, 69.4)	27.1 (13, 48.9)
Age: F = 2.3, *p* = 0.147, Group: F = 0.5, *p* = 0.597, Age × Group: F = 0.1, *p* = 0.932				
17-Hydroxypregnanolone (ng/g)	12	0.922 (0.588, 1.43)	2.54 (1.68, 3.93)	518 (114, 11,000)
	25	1.14 (0.767, 1.69)	0.859 (0.573, 1.27)	40.8 (17.4, 130)
Age: F = 5.8, *p* = 0.025, Group: F = 76.6, *p* < 0.001, Age × Group: F = 2.9, *p* = 0.077; I25 < I12, P > I, P > C, P12 > I12, P12 > C12, P25 > I25, P25 > C25				
Conjugated 17-hydroxypregnanolone [pg/g]	12	3.25 (1.5, 6.77)	5.04 (2.6, 9.78)	275 (87.5, 1160)
	25	5.63 (2.7, 11.9)	2.8 (1.26, 5.84)	82.7 (32.1, 257)
Age: F = 0.5, *p* = 0.506, Group: F = 26.3, *p* < 0.001, Age × Group: F = 1.1, *p* = 0.367; P > I, P > C, P12 > I12, P12 > C12, P25 > I25, P25 > C25				
3α,17α,20α-Trihydroxy-5β-pregnane (ng/g)	12	0.64 (0.379, 1.08)	2.57 (1.59, 4.21)	5.86 (3.54, 9.88)
	25	0.399 (0.247, 0.636)	0.221 (0.132, 0.359)	0.951 (0.565, 1.61)
Age: F = 32.7, *p* < 0.001, Group: F = 10.8, *p* < 0.001, Age × Group: F = 4.5, *p* = 0.023; I25 < I12, P25 < P12, P > I, P > C, I12 > C12, P12 > C12, P25 > I25				
Etiocholanolone (ETIO) (pg/g)	12	53 (45.1, 62.9)	105 (84.9, 132)	654 (333, 7570)
	25	38.9 (33.3, 45.5)	35.8 (30.1, 42.6)	76.3 (63.6, 92.9)
Age: F = 69.5, *p* < 0.001, Group: F = 43.5, *p* < 0.001, Age × Group: F = 6.3, *p* = 0.007; I25 < I12, P25 < P12, P > I, P > C, I12 > C12, P12 > I12, P12 > C12, P25 > I25, P25 > C25				

^1^ C = control group (i.p. CDX solution); ^2^ I = intact control group of animals; ^3^ P = animals treated with PA-G (i.p., 1 mg/kg in CDX).

## Data Availability

Data available within article and Supplementary Materials.

## Supplementary Materials

The following are available online at https://www.mdpi.com/article/10.3390/ph15010049/s1. Table S1: Steroid levels (pM/g) in the hippocampal tissue of rats in the control group of animals treated with cyclodextrin (i.p.), intact animals, and those in the group treated with pregnanolone glutamate (PA-G, i.p. in CDX, 1 mg/kg) 15 min after the application. Table S2: Chemical names, common names, and abbreviations for the steroids studied.
