# Supplementary material for: The Neuroactive Steroid Pregnanolone Glutamate: Anticonvulsant Effect, Metabolites and Its Effect on Neurosteroid Levels in Developing Rat Brains
Source: Pharmaceuticals (Basel). 2021 Dec 30;15(1):49. doi: 10.3390/ph15010049 (PMC8780580; doi:10.3390/ph15010049)
Supplement: Supplementary file 1 [file pharmaceuticals-15-00049-s001.zip › pharmaceuticals-1510945-supplementary.pdf]

pharmaceuticals

Supporting Information

The neuroactive steroid pregnanolone glutamate: anticonvulsant effect, metabolites and its effect on neurosteroid levels in developing rat brain

Eva Kudova [1,*], Pavel Mares [2], Martin Hill [3], Katerina Vondrakova [2,4], Grygoriy Tsenov [2,4,5,*], Hana Chodounska [1], Hana Kubova [2], and Karel Vales [2,4]

[1] Institute of Organic Chemistry and Biochemistry of the Czech Academy of Sciences, Flemingovo namesti 2, 16000 Prague, Czech Republic; hchod@uochb.cas.cz
[2] Institute of Physiology, Academy of Sciences of the Czech Republic, Videnska 1083, 14220 Prague, Czech Republic; pavel.mares@fgu.cas.cz (P.M.); katavondrakova@seznam.cz (K.V.);
hana.kubova@fgu.cas.cz (H.K.); karel.vales@nudz.cz (K.V.)
[3] Institute of Endocrinology, Narodni 8, 11694 Prague, Czech Republic; mhill@endo.cz
[4] Institute of Mental Health, Topolova 748, 25067 Klecany-Prague East, Czech Republic
[5] Department of Neurosciences, Biomedicine and Movement Sciences, University of Verona, Strada le Grazie 8, 37134 Verona, Italy
* Correspondence: eva.kudova@uochb.cas.cz (E.K.); grygoriy.tsenov@nudz.cz (G.T.)

Table of Contents

Table S1. Steroid levels (pM/g) in hippocampal tissue of rats in the control group of animals treated with cyclodextrin (i.p.), intact animals, and in the group treated with pregnanolone glutamate (PA-G, i.p., CDX, 1 mg/kg) 15 min after the application. 2

Table S2. Chemical names, common names, and abbreviations for steroids studied......................4

References 5

Table S1. Steroid levels (pM/g) in hippocampal tissue of rats in the control group of animals treated with cyclodextrin (i.p.), intact animals, and in the group treated with pregnanolone glutamate (PA-G, i.p., CDX, 1 mg/kg) 15 min after the application. %5β = percentage of total significantly increased 5β-steroids in rat hippocampus 15 minutes after PA-G application; Δ_r = steroid increase above the basal levels 15 minutes after the pregnanolone glutamate application; C = controls with cyclodextrin *i .p.* application, I = intact controls, P = PA-G (*i.p.*, CDX); 12, 25 = week 12 and 25 after labor, respectively. The GC-MS/MS based steroid quantification method was described in detail in our recent article.[1]

	Age [Day]	Age × Group			%5β	Δ_r
Steroid		Control	Intact	P	brain	PG/Control
5β-Dihydroprogesterone	12	0.0796 (0.0501, 0.123)	0.294 (0.173, 0.547)	1.51 (0.677, 5.72)	0.07%	19 (111, 856)
(5β-DHP)	25	0.157 (0.0962, 0.263)	0.154 (0.0998, 0.244)	0.299 (0.185, 0.518)	0.09%	1.9 (7.68, 22.8)
		ANOVA: Age: F=1.7, p=0.21, Group: F=11.5, p<0.001, Age × Group: F=4.2, p=0.029; P25<P12, P>I, P>C, I12>C12, P12>C12				
Pregnanolone	12	0.736 (0.509, 1.06)	0.799 (0.575, 1.11)	200 (81.7, 630)	9.47%	272 (111, 856)
(PA)	25	1.23 (0.885, 1.74)	0.334 (0.221, 0.489)	15.8 (9.48, 28.2)	9.30%	12.8 (7.68, 22.8)
		ANOVA: Age: F=6.9, p=0.016, Group: F=131.6, p<0.001, Age × Group: F=7.2, p=0.004; I25<I12, P25<P12, P>I, P>C, P12>I12, P12>C12, I25<C25, P25>I25, P25>C25				
Conjugated	12	0.643 (0.452, 0.896)	1.2 (0.834, 1.72)	128 (58.7, 345)	6.04%	199 (111, 856)
pregnanolone	25	1.93 (1.4, 2.7)	0.746 (0.508, 1.07)	18.9 (11.6, 32.8)	10.83%	9.76 (7.68, 22.8)
		ANOVA: Age: F=0.3, p=0.581, Group: F=115.8, p<0.001, Age × Group: F=11.5, p<0.001; C25>C12, P25<P12, P>I, P>C, P12>I12, P12>C12, I25<C25, P25>I25, P25>C25				
5β-Pregnane-3α,20α-diol	12	0.337 (0.253, 0.449)	1.06 (0.776, 1.48)	26.3 (14.3, 53.9)	1.23%	78.1 (111, 856)
	25	0.182 (0.135, 0.244)	0.0887 (0.0581, 0.128)	2.87 (1.97, 4.31)	1.72%	15.7 (7.68, 22.8)
		ANOVA: Age: F=75.2, p<0.001, Group: F=129.5, p<0.001, Age × Group: F=9.5, p=0.001; C25<C12, I25<I12, P25<P12, P>I, P>C, I12>C12, P12>I12, P12>C12, P25>I25, P25>C25				
Conjugated	12	0.112 (0.0705, 0.174)	0.563 (0.314, 1.05)	11.2 (5.69, 23.7)	0.53%	99.7 (111, 856)
5β-pregnane-3α,20α-diol	25	0.158 (0.101, 0.245)	0.243 (0.157, 0.379)	1.62 (0.971, 2.81)	0.93%	10.2 (7.68, 22.8)
		ANOVA: Age: F=5.9, p=0.024, Group: F=53.7, p<0.001, Age × Group: F=4.3, p=0.026; P25<P12, I>C, P>I, P>C, I12>C12, P12>I12, P12>C12, P25>I25, P25>C25				
17-Hydroxypregnanolone	12	2.76 (1.76, 4.28)	7.59 (5.04, 11.8)	1550 (341, 33000)	73.67%	562 (111, 856)
(17-OH-PA)	25	3.41 (2.3, 5.06)	2.57 (1.71, 3.81)	122 (52, 390)	75.85%	35.8 (7.68, 22.8)
		ANOVA: Age: F=5.8, p=0.025, Group: F=76.6, p<0.001, Age × Group: F=2.9, p=0.077; I25<I12, P>I, P>C, P12>I12, P12>C12, P25>I25, P25>C25				
Conjugated	12	0.00972 (0.00448, 0.0203)	0.0151 (0.0078, 0.0293)	0.822 (0.262, 3.47)	0.04%	84.6 (111, 856)
17-hydroxypregnanolone	25	0.0169 (0.00807, 0.0357)	0.00837 (0.00378, 0.0175)	0.248 (0.096, 0.768)	0.15%	14.7 (7.68, 22.8)
		ANOVA: Age: F=0.5, p=0.506, Group: F=26.3, p<0.001, Age × Group: F=1.1, p=0.367; P>I, P>C, P12>I12, P12>C12, P25>I25, P25>C25				
5β-Pregnane-3α,17α,20α-triol	12	1.91 (1.13, 3.21)	7.65 (4.73, 12.5)	17.4 (10.5, 29.4)	0.74%	9.16 (111, 856)
	25	1.19 (0.735, 1.89)	0.659 (0.393, 1.07)	2.83 (1.68, 4.79)	1.05%	2.39 (7.68, 22.8)
		ANOVA: Age: F=32.7, p<0.001, Group: F=10.8, p<0.001, Age × Group: F=4.5, p=0.023; I25<I12, P25<P12, P>I, P>C, I12>C12, P12>C12, P25>I25				
Etiocholanolone	12	0.183 (0.155, 0.217)	0.36 (0.293, 0.456)	2.25 (1.15, 26.1)	0.10%	12.3 (111, 856)
(ETIO)	25	0.134 (0.115, 0.157)	0.123 (0.104, 0.147)	0.263 (0.219, 0.32)	0.08%	1.96 (7.68, 22.8)

ANOVA: Age: F=69.5, p<0.001, Group: F=43.5, p<0.001, Age × Group: F=6.3, p=0.007; I25<I12, P25<P12, P>I, P>C, I12>C12, P12>I12, P12>C12, P25>I25, P25>C25						
3α,5β-Tetrahydrocorticosterone	12	49.2 (28.1, 79.9)	114 (76.8, 163)	220 (157, 301)	8.12%	4.47 (111, 856)
(3α,5β-THCC)	25	62.2 (39.2, 94)	18.3 (8.72, 32.4)	42.8 (23.8, 70.8)	0.00%	0.688 (7.68, 22.8)
ANOVA: Age: F=18.9, p<0.001, Group: F=3.5, p=0.047, Age × Group: F=6.8, p=0.005; I25<I12, P25<P12, P12>C12						

Table S2. Chemical names, common names, and abbreviations for steroids studied. The first column gives the chemical name, the second column indicate a trivial name accepted for this compound, and the third column shows abbreviation. N/A – abbreviations were not introduced for these compounds.

Chemical Name	Trivial Name	Abbreviation
3α-Hydroxy-5α-pregnan-20-one	Allopregnanolone	ALLO
3α-Hydroxy-5β-pregnan-20-one	Pregnanolone	PA
20-Oxo-5β-pregnan-3α-yl 3-Sulfate	Pregnanolone sulfate	PA-S
20-Oxo-5β-pregnan-3α-yl L-glutamyl 1-ester	Pregnanolone glutamate	PA-G
17α-Hydroxy-20-oxo-5β-pregnan-3α-yl L-glutamyl 1-ester	17-Hydroxypregnanolone glutamate	17-OH-PA-G
(20S)-20-Hydroxy-5β-pregnan-3α-yl glutamate	(20S)-Hydroxypregnanolone glutamate	N/A
3α-Hydroxy-5β-androstan-17-one	Etiocholanolone	ETIO
3α,17α-Dihydroxy-5β-pregnan-20-one	17-Hydroxypregnanolone	17-OH-PA
3α,17α-Dihydroxy-5α-pregnan-20-one	17-Hydroxyallopregnanolone	17-OH-ALLO
3α-Hydroxy-5β-pregnan-20-one	Pregnanolone	PA
(20S)-5β-Pregnan-3α,20-diol	5β-Pregnane-3α,20α-diol	Not introduced
(20S)-5β-pregnan-3α,17α,20-triol	5β-Pregnane-3α,17,20α-triol	Not introduced
5β-Pregnane-3,20-dione	5β-Dihydroprogesterone	5β-DHP
3α,11β,21-Trihydroxy-5β-pregnan-20-one	3α,5β-Tetrahydrocorticosterone	3α,5β-THCC
3β-Hydroxy-5α-pregnan-20-one	Pregnenolone	PE
20-Oxo-pregn-5-en-3β-yl 3-Sulfate	Pregnenolone sulfate	PE-S
20-Oxo-5α-pregnan-3α-yl 3-Sulfate	Allopregnanolone sulfate	ALLO-S
3β-Hydroxy-androst-5-en-17-one	Dehydroepinadrosteone	DHEA
17-Oxo-androst-5-en-3β-yl 3-Sulfate	Dehydroepiandrosterone sulfate	DHEA-S
3β,17β-Dihydroxy-androst-5-ene	Androstenediol	Not introduced
17β-Hydroxy-androst-5-ene-3β-yl 3-Sulfate	Androstenediol sulfate	Not introduced

References

1. Hill, M.; Hana jr., V.; Velikova, M.; Parizek, A.; Kolatorova, L.; Vitku, J.; Skodova, T.; Simkova, M.; Simjak, P.; Kancheva, R.; Koucky, M.; Kokrdova, Z.; Adamcova, K.; Cerny, A.; Hajek, Z.; Duskova, M.; Bulant, J.; Starka, L., A method for determination of one hundred endogenous steroids in human serum by gas chromatography-tandem mass spectrometry. *Physiol. Res.* **2019,** *68* (2), 179-207.
2. Holubova, K.; Chvojkova, M.; Krausova, B. H.; Vyklicky, V.; Kudova, E.; Chodounska, H.; Vyklicky, L.; Vales, K., Pitfalls of NMDA Receptor Modulation by Neuroactive Steroids. The Effect of Positive and Negative Modulation of NMDA Receptors in an Animal Model of Schizophrenia. *Biomolecules* **2021,** *11* (7).
